# Everybody's Page

**Published:** 1890-09-27

**Authors:** 


### Everybody's Page.

The report of the Education Department is encouraging from the sanitarian's point of view. The children are reported to be much better physically for going to school. The benefits which the Country Holiday Fund confers, together with the good derived from wholesome cheap meals, and large open playgrounds where the children get a chance of development, are clearly visible. It is gratifying to know this. The sanitarian, poor thing, has to stand a good deal of ridicule; he is considered by some to be the 19th century alarmist; but ridicule cannot upset solid facts, and when we hear such as these about the children's health, we are inclined to think that it is the sanitarian's turn to laugh.

Sir Charles Cameron, in the *British Medical Journal*, tells of ten people who ate oysters at a luncheon party in Dublin last week, and who have everyone been suffering from nausea, vomiting, diarrhœa, and abdominal pain; the oysters seemed to be in good condition. Sir Charles Cameron has previously pointed out the fact that oysters growing anywhere near a place to which sewage has access must very often contain sewage in their juice, and he has found this in the case of oysters gathered near Dublin. It is would be well to inquire the origin of these fatal delicacies. Oysters are an expensive luxury; they may prove a very dangerous one. We hear much of the necessity of meat inspection, and having achieved that successfully, the next thing ought to be fish inspection.

The Aylesbury Dairy Company owes a debt of gratitude to "Idiotes," who writes in the *Times* to praise the vigilance and care they exercise in order to avoid the spread of any infectious disease. A case of measles occurring in his house, he told the milkman of it, who said, "Oh! then I must not call any more, you must be served special"—which, being interpreted, meant that the milk would be delivered by a special messenger who never enters the dairy, and whose cans are all especially cleansed and disinfected. Now, what better advertisement could the Aylesbury Company have? But, all the same, it shows the general public that when we urge them to be careful where they get their milk from, there is some reason in it, and that paying a little more to Welford or the Aylesbury, or any other well-known place, may prove cheaper than paying a penny a quart less.

Yet another invention for smoke prevention. Mr. Greaves, of Manchester, claims the merit of the most recent apparatus. The invention was first tried last October, and met with such success that there are now 160 boilers in and around Manchester all working on this system. The very mention of smoke or its prevention makes us feel dismal. Pictures of London in the winter, and London fogs, and the pitch darkness at our one o'clock dinner loom before us, and we find ourselves wondering whether Londoners will go on living half their winters through in semi-darkness, choked by innumerable bits of coal dust. We feel perfectly envious of Manchester, and we wish Mr. Greaves would by a little persuasion in London see what he could do not only for the manufactories, but for the ordinary domestic chimney as well!

The London Missionary Hospital at Pekin has proved its good work by the popularity in which it is held by the natives. The accommodation for in-patients has become quite inadequate for the applications. The war which the hospital wages against opium smoking is considerable. Some of the reasons why the patients desire to break off the habit are instructive. For instance: Want of money, 39 patients —an excellent reason; loss of time or of character, 61; injury to health, 10; home influence, 6; and Christian influence, 3.

One of the great arguments used against temperance by those who do not care to practice it is that neither mental nor physical labour can be carried on long without some sort of stimulant. Mr. Caine fought valiantly for the Temperance League at Sleaford, Lincolnshire, last week, combatting this argument as an entire fallacy. For proof of this statement that not the slightest benefit accrued from alcoholic beverages he mentioned the expeditions of General Wolseley in the Red River when for seven months the troops were without stimulant, also the expeditions to the North Pole. There are 15,000 teetotallers in the British army, and they certainly deserve admiration for sticking to their resolutions whilst there are so many around them who drink. For temperance work it must be a case of do as I do and not only as I say, and those who take the pledge are the only people who meet with any success in reducing the drink evil. But, as we have said before, the work of reform lies in starting the young with sober ideas, and though the scornful may sneer and be sceptical, we are convinced that the numerous temperance leagues and guilds are mustering no slight aid against the greatest curse of England.

We notice in the daily papers that the Rev. F. Lawrence, the hon. secretary of the Church of England Burial Association, publishes letters which he has received from Mr. Spurgeon and the Bishop of Worcester respecting the much-talked-of burial reform. Mr. Spurgeon does not write very hopefully when he says that when the association has conquered existing prejudices another host of extravagances will spring up. This is rather anticipating evil. The Bishop of Worcester deprecates the wasting of money on funerals by both poor and rich alike. "The poor," says he, "waste money which they much need for other things, and the rich often encourage the false notion that they honour their departed friends by the vain pomp of costly funerals." This is quite true; there are few things more revolting than the "pomp" of a rich man's burial. Pounds and pounds are often expended on trappings for moth and rust to corrupt. The procession in the streets acts as a bad example of extravagance to the passers-by, and our honest grief is turned into a spectacle of painful display. The chief aim of the ancient Egyptians' life was to be well buried; it was a high ambition certainly, but it is scarcely noble of the rich Englishman to teach by his example his poorer neighbour that to be "well buried" is a respectable thing. The poor man often sacrifices a great deal to bury one of his relatives "well." Mr. Spurgeon describes such funerals as "customs which oppress the fatherless and widow, by demanding of them an expenditure which they cannot afford." Inexorable custom so far precludes the "reform" being very great, but we applaud and respect the example of any who aims at demolishing this remnant of heathenism.

